# Delivery of a peptide-drug conjugate targeting the blood brain barrier improved the efficacy of paclitaxel against glioma

**DOI:** 10.18632/oncotarget.12708

**Published:** 2016-10-17

**Authors:** Ying Li, Xuemin Zheng, Min Gong, Jianning Zhang

**Affiliations:** ^1^ Tianjin Neurological Institute, Tianjin Medical University General Hospital, Tianjin, China; ^2^ Tianjin Institute of Pharmaceutical Research, Tianjin, China; ^3^ School of Pharmacy, Tianjin Medical University, Tianjin, China; ^4^ Department of Oncology, University of Oxford, Oxford, UK

**Keywords:** blood brain barrier, low-density lipoprotein related protein-1, brain glioma, cell penetrating peptide, paclitaxel

## Abstract

The challenge of effectively delivering therapeutic agents to the brain has created an entire field of active research devoted to overcoming the blood brain barrier (BBB) and efficiently delivering drugs to the brain. Angiopep-2 can trigger transcytosis and traverse the BBB by recognizing low-density lipoprotein related protein-1 (LRP-1) expressed on the brain capillary endothelial cells. Here, we designed a novel strategy for the delivery of drugs to the brain. The novel drug delivery system was a combination of a receptor-targeting ligand, such as low-density lipoprotein related protein 1, and a cell-penetrating peptide (CPP). It was hypothesized that this conjugate will enhance the delivery of associated therapeutic cargo across the BBB and increase the permeability of a solid tumor. Our findings indicate that the combination of these two agents in a delivery vehicle significantly improved translocation of small molecules (paclitaxel) into the brain compared to the vehicle treatment, which contained only receptor-targeting ligand. The application of this strategy could potentially expand the horizons for the treatment of central nervous system disorders.

## INTRODUCTION

Central nervous system (CNS) diseases, brain tumors, neurological disorders, and neurodegenerative diseases, have become main threats to human health, and it was estimated that the CNS diseases will reach 14% of all global diseases by 2020 [1]. However, the blood−brain barrier (BBB) poses challenges for the development of drugs for diseases of the central nervous system (CNS) due to its tight junctions and lack of fenestration, which prevent drugs from reaching the site of disease [2]. Novel brain-targeted drug delivery strategies have attracted extensive attention for their ability to deliver drugs across the BBB and increase the survival rate of glioma patients [2, 3].

Receptor-mediated transcytosis (RMT) has been emerging as a successful strategy to conquer the BBB because brain capillary endothelial cells express many different receptors, including transferrin receptors, low-density lipoprotein-related protein 1 (LRP-1), and nicotine acetylcholine receptors [4, 5]. The endogenous ligands trigger receptor-mediated transcytosis after binding to specific receptors [6]. In addition, those ligands have inspired the development of novel brain-targeted drug delivery strategies, which possess great potential for improving the diagnosis and treatment of CNS diseases [3, 7].

Peptide-based ligands have been widely used to facilitate brain-targeted drug delivery following structural design and phage display screening. Angiopep-2, which was developed as a specific ligand for LRP-1, is a 19-mer peptide derived from the human Kunitz domain [8]. Angiopeptide-2 possesses extraordinary potential for stimulating brain-targeted drug delivery [9]. Angiopep-2 transport and accumulation in brain endothelial cells can be blocked by α2-macroglobulin, a specific ligand for LRP-1, indicating that Angiopep-2 facilitates brain-targeted drug delivery through LRP-1-mediated transcytosis [10]. Low-density lipoprotein receptor-related protein-1 (LRP-1), a member of the low-density lipoprotein receptor family, is highly expressed on the BBB [11, 12]. LRP-1 mediates the transcytosis of multiple ligands across the BBB, such as lactoferrin, melanotransferrin and receptor-associated proteins. More importantly, LRP-1 is also over-expressed in human glioma cells [7]. This indicates that LRP-1 is a potential target moiety for drug delivery systems, as it could be used for targeting not only the BBB but also glioma. A conjugate of angiopep-2 and 3 molecules of paclitaxel, known as ANG1005 in a phase II clinical trial, can penetrate into the brain compartment by targeting LRP-1 [8, 9]. *In vivo* and *in vitro* data demonstrate that the uptake rate of GRN1005 in the brain is 86-fold greater than paclitaxel alone.

However, these specific ligands have high affinity only for targeted receptors and are usually not efficient enough to enhance solid tumor penetration. In this study, cell penetrating peptides, a class of diverse short peptides widely used for siRNA, proteins and small molecule drugs were conjugated with Angiopep-2, allowing for increased drug permeability in the whole brain and gliomas [13]. Herein, we report that the novel drug targeting system, which is comprised of Angiopep-2, cell penetrating peptides (TAT) and paclitaxel increased drug distribution in an intracranial model of glioblastoma in nude mice.

## RESULTS AND DISCUSSION

### Cellular uptake

**Figure 2:**
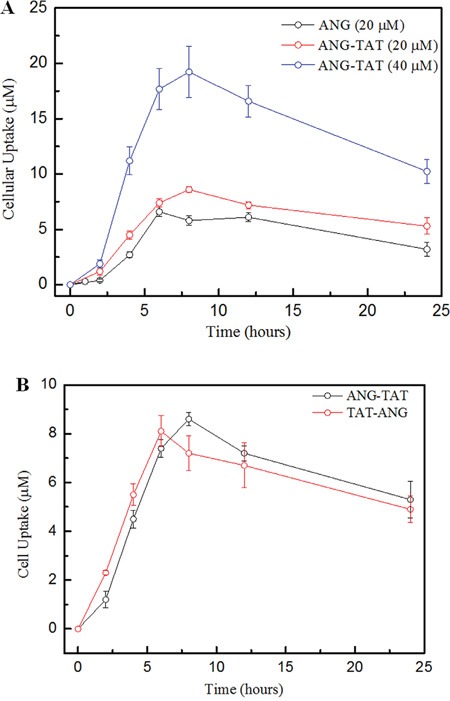
Biotin labelled peptides uptake into U87 human glioma cells **Panel A.** Uptake of biotin labeled ANG at various concetrations follwoed 2, 4, 8, 12 and 24 hours incubation in U87 human glioma cells. **Panel B.** Uptake of biotin ANG-TAT and biotin TAT-ANG (40 μM) measured at 24 hours after incubation in U87 human glioma cells. Uptake was quantified upon the concentration of labeled biotin. Data are expressed as the mean±s.e.m. of quadruplicate experiments (P<0.05).

To examine whether conjugation with the TAT peptide increased the cell penetrating activity of Angiopep-2, the amount of intracellular peptides was quantitatively determined. U87 glioblastoma cell uptake was significantly higher for ANG-TAT than ANG (Figure 2A). Additionally, the uptake of ANG-TAT by U87 glioblastoma cells was obviously time and concentration dependent.

The TAT fragment also improved the cell penetrating activity of ANG when the TAT fragment was attached to either the N- (TAT-ANG) or C-terminus (ANG-TAT), as shown in Figure 2B. However, the TAT-ANG peptide failed to cross the BBB as measured by an additional brain extraction assay (data not shown).

### Brain uptake of ANG-TAT

**Figure 3:**
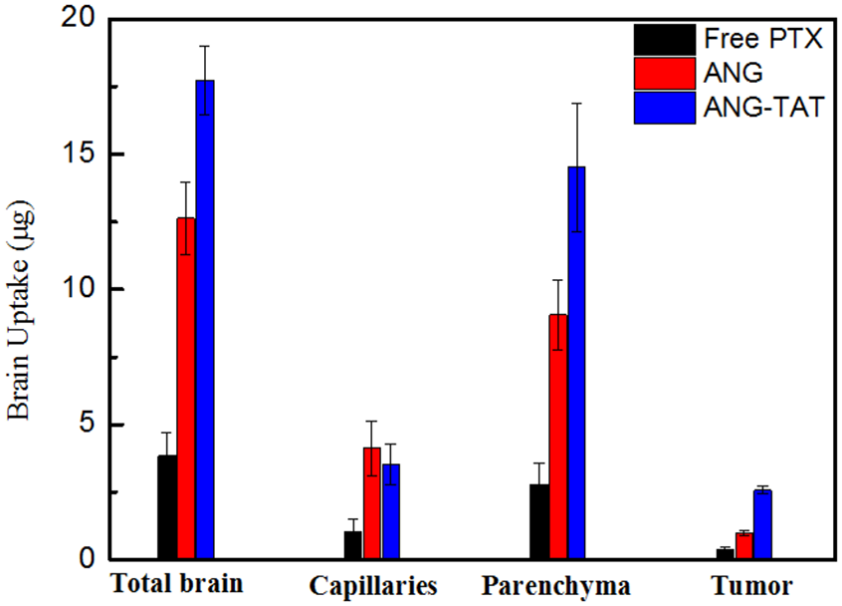
Brain uptake of ANG-TAT measured by in situ brain perfusion CD-1 mice were perfused with biotin labeled ANG-TAT (50 μM) for 5 min. After perfusion, brain capillary depletion was performed on the mice right brain hemispheres. The amount of labeled biotin associated with total brain homogenate, the brain capillary fraction and with the parenchyma was evaluated.

To quantify the peptides in the brains of tumor-bearing mice, U87 glioblastoma cells were implanted intracerebrally via unilateral stereotactic inoculation. The cerebrovascular permeability of ANG-TAT was then analyzed. To perform this experiment, biotin labeled peptides were administered to sedated mice via tail vein injection. Quantitation of biotin in the brains of mice sacrificed at various times following peptide administration was used to evaluate the brain permeability of biotin ANG and biotin ANG-TAT. The right brain hemisphere (tumor implanted) was isolated on ice, and capillary depletion was performed. In addition, peptide uptake in the left brain hemisphere was monitored to evaluate the distribution of the peptides in total brain. Aliquots of homogenates, supernatants and pellets were taken to measure the content of biotin labeled molecules. The results of this analysis indicated that the peptides diffused in brain tissue immediately after their administration. Biotin ANG-TAT exhibited significant increases in tumor permeability compared to biotin ANG (Figure 3). The amount of biotin ANG-TAT extracted from tumor tissue was 1.8-fold higher than biotin ANG and 5.6-fold higher than free paclitaxel alone (P<0.05). These results suggested that the TAT fragment improved the cell penetrating activity of the ANG peptide, especially for solid tumors.

### *In vivo* efficacy of ANG-TAT-PTX treatment in mice bearing U87 glioblastoma brain tumors

**Figure 4:**
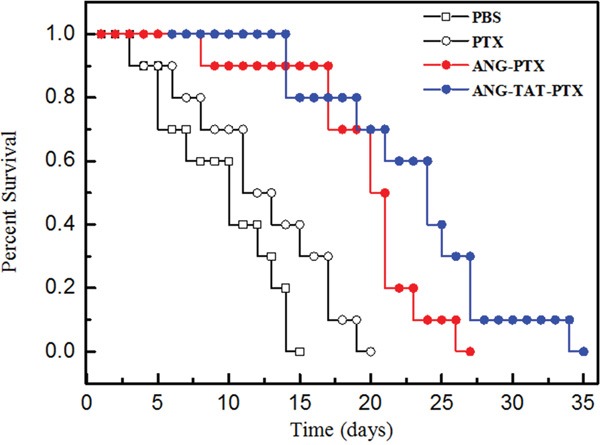
Anti-tumor efficacy of conjugate on human tumor bearing mice Xenograft mice bearing U87 human glioma cells were treated with the ANG-TAT-PTX, ANG-PTX and free paclitaxel, respectively. Survival time was recorded in days after tumor injection. Results indicated that the ANG-TAT-PTX (•) exhibited an improved survival time compared to free paclitaxel (○) in U87 human glioma cells bearing mice, P<0.05 (n=10).

It would be expected that ANG-TAT-PTX treatment in mice with intracranial tumors could confer a survival advantage when compared to treatment with ANG-PTX. To assess this possibility, U87 glioblastoma cells were intracranially implanted into mice. Then, 12 days after tumor cell implantation, mice were then treated with 15 mg/kg vehicle, ANG-PTX, or ANG-TAT-PTX every three days. Mice (*n* = 10) in the survival studies were monitored daily for body weight loss and were euthanized if a weight reduction of greater that 20% from the maximum weight was observed. The results shown in Figure 4 revealed that the administration of ANG-TAT-PTX significantly increased the survival rate of experimental mice compared to mice treated with ANG-PTX or PTX alone.

Thereafter, cellular MTT assay was used to assess the antitumor activity of the ANG-TAT-PTX and to ascertain the safety evaluation of peptides alone (ANG and ANG-TAT). The data of MTT assay indicated the ANG-TAT-PTX resulted in significant loss of cell viability in human U87 glioblastoma cell compared to that of ANG-PTX (Table 1). In addition, the MTT results indicated that the safety of ANG and ANG-TAT were qualified as drug controlled release system.

**Table 1:** MTT assay of ANG-TAT-PTX in tumor cells, compared with that of ANG-PTX

	U87 glioblastoma cells				
Time	Free PTX	ANG-PTX	ANG	ANG-TAT-PTX	ANG-TAT
24h	20.53±2.21	33.21±3.32	8.44±0.86	31.65±3.28	10.19±1.19
48h	42.36±2.35	50.24±4.75	14.65±1.29	73.53±6.45	16.53±1.73

**Condition:** The cells (1 × 10^5^cells/100 μl/well) were seeded in 96-well plates at 37°C and 5% CO2. The aqueous solutions of samples were added in the culture medium following MTT assay protocol.

**Legend:** In MTT assay, the ANG-TAT-PTX exhibited an improved anti-tumor activity compared to that of ANG-PTX, suggesting the presence of TAT fragment increased the cellular uptake of PTX.

## MATERIALS AND METHODS

### Peptides

**Figure 1:**
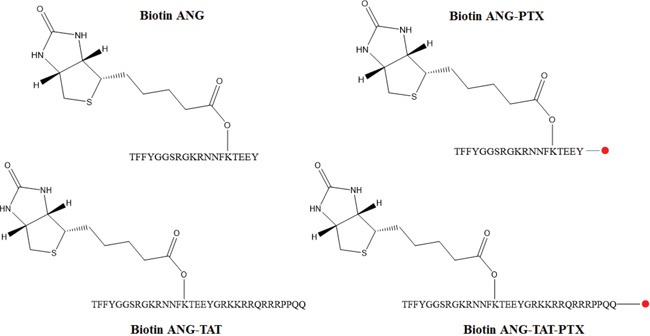
Chemical structure for peptides in this article The biotins labeled peptides (ANG and ANG-TAT) were synthesized using solid phase peptide synthesis protocol and the purified product was then reacted with free paclitaxel (●). The final product was purified by RP-HPLC with XBridge C18 silica column and ascertained by HPLC-MS.

Angiopep-2 (ANG: TFFYGGSRGKRNNFKTEEY), Biotin labeled Angiopep-2 (biotin ANG: TFFYGGSRGKRNNFK[Biotin]TEEY) and biotin labeled Angiopep-2—TAT (biotin ANG-TAT: TFFYG GSRGKRNNFK[Biotin]TEEYGRKKRRQRRRPPQQ). Paclitaxel (PTX) was covalently conjugated with ANG (ANG-PTX) and ANG-TAT (ANG-TAT-PTX) at the C-terminus (all structures in Figure 1), respectively. All peptides used in this study were purchased from Sangon Biotech Co. (Shanghai; HPLC-purified; purity>95%, identified by MS). The freeze-dried peptides were weighed and dissolved in saline to create 10 mg/ml stock solutions for further analysis. Conjugation of PTX with ANG and ANG-TAT was performed by Demochem Co. (Shanghai, China). All other chemicals, if not indicated specifically, were obtained from Sigma-Aldrich.

### Cell culture

The human U87 glioblastoma cell line was purchased from the American Type Culture Collection and cultured in a humidified atmosphere of 5% CO_2_ in modified Eagle's medium containing 10% FBS, 2 mM glutamine, 100 U/ml penicillin, and 100 mg/ml streptomycin.

### Animals

Male CD-1 nude mice (6–8-weeks old) from the Shanghai Laboratory Animal Co. and the China Academy of Sciences were maintained in a pathogen-free environment and handled in accordance with guidelines from the Tianjin Medical University Council on Animal Care.

### Cell uptake

U87 cells were seeded into 12-well plates at a density of 10^5^ cells per well. After 24 h incubation, the cells were incubated with biotin ANG (20 μM) and biotin ANG-TAT (20 μM and 40 μM) peptides in DMEM supplemented with 10% FBS at 37°C for 4 h. The efficiency of cellular biotin ANG and CPP labeled biotin ANG-TAT uptake were identified by measuring the intracellular concentration of biotin using the biotin quantitative kit from Elabsciences, Inc. (Shanghai, China). In brief, the cells were collected and lysed, and then the biotin measuring assay was performed following the manufacturer's protocol. The samples were initially incubated with Avidin-HRP for 30 minutes, and the OD_450 nm_ was monitored after reaction termination.

### Brain extraction of biotin ANG and biotin ANG-TAT

Biotin ANG and biotin ANG-TAT (50 μg/kg body weight) were injected into the tail vein of CD-1 nude mice carrying intracerebral implants of U87 glioblastoma cells. Fifteen minutes after injection the mice were killed, and the brains were surgically removed. The brain tissues were dissected and maintained in cold saline containing protease inhibitors until processing. To compare normal and tumor brain tissue, the right hemisphere (implanted hemisphere) was compared with the left hemisphere (non-implanted hemisphere). To extract biotin ANG and biotin ANG-TAT, tissues were mechanically homogenized in homogenization buffer (0.1 M Tris–HCl, pH 5.0, 50 mM sucrose, 0.003% Tween-80, and protease inhibitor cocktail) on ice. Acetonitrile (ACN) 100% was added to a final concentration of 65% (v/v). The mixture was stirred for 10 min in a cold room and then centrifuged at 3000 r.p.m. for 5 min. Acetonitrile supernatants were collected and dried in a rotative evaporator and finally freeze-dried. The concentration of biotin ANG and biotin ANG-TAT in the samples was determined using biotin quantitative kit from Elabsciences, Inc (Shanghai, China).

### Intracranial model of U87 glioblastoma in nude mice

The U87 cell line was used to assess peptide uptake in an animal model of glioma. Cells suspension were prepared at a final concentration of 1×10^4^ cells/ml in PBS and kept on ice until injection. To perform intracerebral stereotactic implantation of U87, male CD-1 nude mice were deeply anesthetized subjected with isoflurane. The scalp was then swabbed with iodine and alcohol, and the skin was cut. A 10-μl syringe was used to inoculate 5 μl of U87 cell suspension into the corpus striatum of the right hemisphere (3.0 mm deep; 1 mm anterior and 2 mm lateral to the bregma). The skin was sutured with three knots, and then tissue glue and local analgesia were applied to the area. Further brain extraction assays were performed in the animals 10 days after tumor implantation.

### Treatment of U87 brain tumor-bearing mice

Intracerebral U87 tumors were initiated via unilateral stereotactic inoculation of 1 × 10^6^ U87 glioblastoma cells into the brains of mice. One hour before surgery, mice received a subcutaneous injection of buprenorphine (0.1 mg/kg). Mice were placed in a stereotactic apparatus (Kopf) and maintained under anesthesia with isoflurane to perform tumor cell inoculation. A burr hole was drilled 1.5 mm anterior and 2.5 mm lateral to the bregma. Cells suspended in 5 μL of serum free cell culture medium containing 0.5% methylcellulose were injected using a Hamilton syringe at a depth of 3.5 mm over a 5-minute period. Drug treatments began 12 days after tumor cell inoculation. Both ANG-PTX and ANG-TAT-PTX were administered at final concentrations of 15 mg/kg body weight in sodium acetate buffer (150 mmol/L NaCl, 5 mmol/L NaOAc, pH 5) every 3 days. Body weights and clinical signs of disease progression were monitored daily. During the experimental period of 20 days, the survival time was recorded as days after tumor injection. The mean and median survival times and the statistical significance of the results were determined using a two-tailed Wilcoxon's signed-rank test. Repeated experiments were pooled for statistical analysis.

### MTT assay

The cytotoxicity of ANG, ANG-TAT, ANG-PTX and ANG-TAT-PTX were determined using the 3-[4,5-dimethylthiazol-2-yl]-2,5-diphenyl tetrazolium bromide (MTT) assay. Human U87 glioblastoma cell (1×10^5^ cells/100 μl per well) were cultured in 96-well plates at 37°C in 5% CO2. Aqueous solutions of the samples were dissolved in culture medium at final concentrations of 0.1, 0.5, 1, and 2.5 mg/mL. After incubation for 24 and 48 hours, the MTT solution (in 2 mg/mL phosphate-buffered saline) was added, the plates were incubated for 4 hours, and the cells were lysed with 50% N, N-dimethylformamide containing 20% sodium dodecyl sulfate (pH 4.5). Absorbance at 570 nm was measured for each well using a SpectraMax M5 (Molecular Devices, Sunnyvale, CA USA). The absorbance of control cells was taken as 100% viability, and the values of the treated cells were calculated as a percentage of the control.

### Statistical analysis

Data are expressed as the mean ± SD or SEM. Statistical analyses were performed using student's t test when one group was compared with the control group. ANOVA was used when two or more groups were compared. For survival studies, statistical analysis was performed using a two-tailed Wilcoxon's signed-rank test. P < 0.05 was considered statistically significant.

## CONCLUSION

Angiopep-2 peptide has been shown to be able to cross the blood–brain barrier (BBB) by receptor-mediated transcytosis via low-density lipoprotein receptor-related protein 1 (LRP1). Interestingly, recent evidences also showed an increased uptake of ANG1005 into tumor tissue of mice bearing brain metastases of the aggressive human breast cancer cell line MDA-MB-231 [6, 9].

In this study, Angiopep-2 was conjugated to cell penetrating peptides to improve its cell permeability in solid tumors. This novel drug delivery system showed increased cellular uptake and solid tumor penetrating efficiency. Therefore, conjugation of Angiopep-2 with cell penetrating peptides could serve as an efficient glioma drug delivery system.
